# Predicting Visibility of Aircraft

**DOI:** 10.1371/journal.pone.0005594

**Published:** 2009-05-20

**Authors:** Andrew Watson, Cesar V. Ramirez, Ellen Salud

**Affiliations:** NASA Ames Research Center, Moffett Field, Virginia, United States of America; University of Sydney, Australia

## Abstract

Visual detection of aircraft by human observers is an important element of aviation safety. To assess and ensure safety, it would be useful to be able to be able to predict the visibility, to a human observer, of an aircraft of specified size, shape, distance, and coloration. Examples include assuring safe separation among aircraft and between aircraft and unmanned vehicles, design of airport control towers, and efforts to enhance or suppress the visibility of military and rescue vehicles. We have recently developed a simple metric of pattern visibility, the Spatial Standard Observer (SSO). In this report we examine whether the SSO can predict visibility of simulated aircraft images. We constructed a set of aircraft images from three-dimensional computer graphic models, and measured the luminance contrast threshold for each image from three human observers. The data were well predicted by the SSO. Finally, we show how to use the SSO to predict visibility range for aircraft of arbitrary size, shape, distance, and coloration.

## Introduction

### The need for visibility metric in aviation

In the United States National Air Space (NAS), Federal Aviation Regulations require that pilots in visual meteorological conditions (VMC) be able to “see and avoid” other aircraft, in order to assure safe separation. However, little research has been done to determine the feasibility or effectiveness of this requirement [1]. The widespread adoption by commercial aircraft of TCAS, which incorporates transponders that warn of nearby aircraft, has reduced reliance on see-and-avoid. But the rule still applies under VMC, and most general aviation aircraft do not have TCAS. Mid-air collisions remain a serious problem for small aircraft in general aviation [2].

Further, the see-and-avoid requirement has become a significant barrier to the introduction of unmanned aircraft (UA) into the NAS [3]. The problem is twofold: first, the visibility of a UA to piloted aircraft is unknown. Second, the UA is piloted from a remote location, usually via video from an on-board camera, and visibility of piloted aircraft to the UA pilot is also unknown.

Visual detection of aircraft is also crucial in military operations. Of two opposing fighter pilots, the one who first detects the other is most likely to prevail. And in this context, visibility may depend upon the optical qualities of devices such as visor and canopies.

Visibility of aircraft will depend upon their size, shape, distance, and coloration, as well as upon atmospheric and lighting conditions, but collection of human empirical data for all of the possible variations among these quantities is not practical. An alternative approach is to develop a general model for visibility of aircraft targets to a human viewer. Such a model would enable rapid evaluation of aircraft visibility in particular contexts. In particular, it could be used to assess the limitations of the see-and-avoid principle under diverse conditions, in order to provide better protective measures.

A visibility metric would also have important applications in evaluation of military operations and procedures that depend upon visual detection of other aircraft, vehicles, missiles, and targets. These applications could extend beyond aviation, to ground and naval operations as well.

### Previous research

Despite the centrality of the “see and avoid” doctrine, there have been few experimental or theoretical studies of the practice. Here we review the few attempts that have been made to measure or model the see and avoid process, focusing on those studies that address the visual detection component of the problem. A comprehensive analysis of the see and avoid problem must consider at least the following aspects: visual search, field of view, speed and angle of approach, and detectability of the target [4]. Here we are concerned with only the last of these elements (see [2] for discussion of visual search).

Howell [5] carried out a field study in which pilots attempted to detect another aircraft (DC-3) approaching on a collision course. Over various conditions, the average distance at which detection by the pilot occurred (“detection distance”) was from 5.5 to 8.7 km. Of greater relevance to this study, the subject aircraft also carried an experimenter who knew exactly the approach angle of the target aircraft, and “kept constant vigil with his naked eye” until he detected the intruder aircraft. This “threshold distance”, over the same conditions, averaged from 17.3 to 23 km, about three times larger than the detection distance. We will return to these results later in this paper. Analyzing these data, Graham and Orr concluded that see and avoid failures were due primarily to failure to detect the target [1]. No attempt was made to predict aircraft visibility.

One early report that did attempt such predictions was that of Harris [6], [7]. Digitized photographs of three scale aircraft models (DC-3, DC-8, and 737) in the three poses (0, 45, and 90 deg from nose-on) were convolved with a “summative function,” designed to represent the spatial summation properties of the human visual system. Various ranges were simulated by scaling the size of the aircraft image. The calculations also took into account exponential contrast attenuation by atmospheres of different meteorological ranges. The exercise was repeated for three aircraft at three different orientations. This allowed calculation of the range at which each of the aircraft could be detected under specified atmospheric conditions. For example, the DC-3, at 45° orientation, would be detected at 18 km with no atmospheric attenuation. While a remarkable advance for its time, the model could only make predictions for the particular scaled aircraft models and poses employed. The present effort is quite similar in spirit and methods, though we use digital aircraft models and take advantage of a more advanced model of human vision.

Andrews [4], [8] analyzed actual approaches between a subject and an interceptor aircraft. He developed a mathematical model for probability of acquisition that included a wide variety of factors, such as search, field of view, speed of approach, and aircraft detectability. Detectability was assumed to be simply proportional to the product of target solid angle and contrast, with the proportionality constant estimated from the data for different aircraft. Simplifications were adopted to approximate the area of complex aircraft shapes viewed from arbitrary angles. While the model provided a good fit to the data, it is unclear how predictions would be made for other aircraft with different shapes or contrasts.

One long-established method for calculating visibility of targets in technical applications is the so-called Johnson metric and its successors such as NVTherm and TTP [9]). These metrics consider the modulation transfer function of the imaging system, and the contrast sensitivity function of the human observer, and the size and contrast of the target, to compute an effective number of visible “cycles on target.” These values can be converted into a probability of success in a given task with the aid of calibration factors (N50, V50). However, these calibration factors must be determined empirically for each task (e.g. detection, or discrimination of a class of vehicles, or identification of a specific vehicle type). Since determination of these factors is laborious and subject to experimental error, this limits the utility and generality of this approach.

Some research has addressed the relationship between properties of human vision and aircraft detection [10], [11]. That work showed a correlation between individual contrast sensitivity and performance in detection of aircraft or ground targets. However it did not provide a metric to predict visibility of aircraft.

### Aircraft images and contrast thresholds

Viewed from a distance, an aircraft projects an image upon the eye of an observer. In analyzing the response of the eye to a visual image, we usually express the dimensions of the image in degrees of visual angle: the angle subtended by a triangle with one vertex at the eye and the other two enclosing the object. We also often convert the brightness (luminance) of the object to contrast: the difference from the background expressed as a proportion of the background. After these conversions, the aircraft image becomes a two-dimensional distribution of contrast over space measured in degrees. A fundamental question in vision science is when such a distribution becomes visible. For each distribution, we can consider varying its contrast, without varying its shape. We can then define the contrast threshold as the contrast at which the distribution can just be detected. From measurements of the contrast threshold of many different distributions, a few key principles have emerged. First, threshold varies as function of the spatial frequency of the distribution. Spatial frequency is the frequency of a sinusoidal variation in contrast across space, but intuitively corresponds to the fineness of detail. High spatial frequencies (fine details) are more difficult to see (require more contrast) than do middle frequencies (medium detail). This variation is captured in a function called the contrast sensitivity function, which expresses sensitivity (the inverse of threshold) as a function of spatial frequency. Second, not surprisingly, patterns are more visible when they are close to the point of fixation. The third, less obvious principle is that spatial integration of the distribution is non-linear. Rather than pooling the contrast, the brain pools something close to the contrast energy of the distribution. Just these three principles, accurately applied, can account for much of the variation in contrast thresholds for a wide variety of patterns [12]. We have combined these principles into a simple metric called the Spatial Standard Observer (SSO), that will be described in more detail below [13].

Since aircraft images are simply another class of contrast patterns, it is reasonable to suppose that their contrast thresholds can be predicted by the SSO. If that proves true, then the SSO will provide a valuable tool to predict visibility of arbitrary aircraft under arbitrary viewing conditions. As we will show, it can also be used to predict the distance at which an aircraft can be detected.

In this analysis we have ignored the dimension of time. The time dimension will be important when aircraft are near to the observer, or move against a patterned background, or display blinking lights at nighttime. But for a broad range of important conditions — distant aircraft, daytime, homogeneous sky background — time plays little role. Under such conditions, the aircraft are effectively stationary.

### Spatial Standard Observer

**Figure 1:**
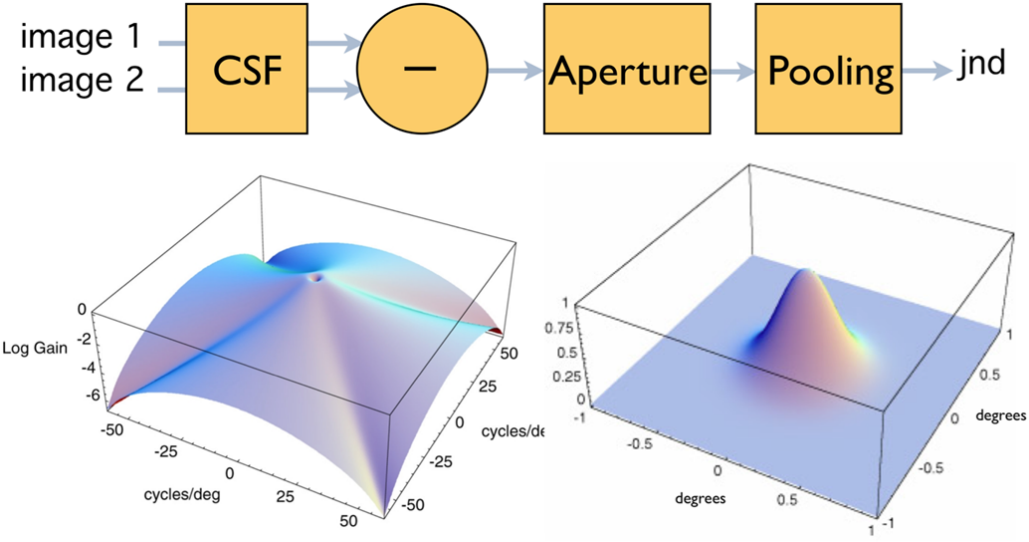
Overview of the Spatial Standard Observer. The upper diagram shows the sequence of operations. The lower left shows the CSF in the spatial frequency domain, and the lower right image shows the Gaussian spatial aperture in the space domain.

The Spatial Standard Observer (SSO) is a new metric that computes contrast thresholds for arbitrary grayscale images [12], [13]. An outline of the SSO is shown in Figure 1. The input to the metric is the pair of images that are to be discriminated. The grayscale of the pixels in each image are proportional to luminance. The two images pass through a contrast sensitivity filter (CSF) and are subtracted. The difference is then multiplied by a Gaussian spatial aperture, and the result is pooled using a Minkowski summation (the pixels are rectified, raised to a power *b*, summed over both spatial dimensions, and the result raised to the power 1/*b*). Output of the metric is in just-noticeable difference (JND) units. For a detection task, one of the images is a uniform luminance, and the other contains a target embedded in a uniform luminance, and threshold occurs when the output has a value of 1 JND.

The SSO is based on a set of data collected in a research project called ModelFest (Carney et al., 1999; Carney et al., 2000; Watson, 1999). The data set consisted of contrast thresholds for 43 stimuli from 16 observers in 10 labs. In the present experiment, we will measure a subset of those thresholds to test agreement between our observers and those of the ModelFest project.

### Experimental Approach

In principle, the SSO could provide a means for estimating the distance at which an aircraft of a specific type, size, and coloration could be seen, whether by a ground observer, or by a pilot in another aircraft, or by a UA pilot on the ground viewing other craft remotely from a UA-mounted sensor. But before this metric can be entrusted with these tasks, we must verify that it can indeed predict the visibility of images of aircraft.

The purpose of this experiment is to first measure the visibility of aircraft images using human observers, and then to compare those results with values predicted by the SSO. To measure visibility, we have obtained contrast thresholds for aircraft targets. A contrast threshold is a measure of the smallest target contrast that can be detected reliably.

To create aircraft images that vary in size, shape, and orientation, we have used 3D computer graphic models of aircraft. These can be posed in a desired position with appropriate lighting, and rendered into 2D images, which can then be used in the experiment. This approach, as opposed to the use of photographs of real aircraft or scale models, is inexpensive and flexible. We can easily acquire a wide range of aircraft, and can pose them in any desired position, lighting, or distance, and with any desired coloration. Although these models lack some detail and “realism” that might be evident in photographs, and lack both color and markings, it should be noted that we are interested in these images when they are at the threshold of visibility, at which point the realism of the image is unlikely to be evident.

It should also be recognized that there are an infinity of possible views of aircraft, from among which we have selected only 38. We emphasize that this experiment is not intended to provide a comprehensive database of thresholds for all possible views of all possible aircraft. Rather, the two purposes of this experiment are 1) to provide a small set of empirical thresholds for targets whose images are likely to span the dimensions of interest (size, brightness, shape, orientation), and 2) to test whether the SSO is able to make accurate predictions of those thresholds. If validated in this way, the SSO could then be used to predict thresholds for arbitrary views of arbitrary aircraft.

## Methods

### Ethics Statement

Informed consent was obtained from all human observers in this project, and the research protocol was reviewed and approved by the Human Research Institutional Review Board of NASA Ames Research Center.

### Stimuli

Stimuli were presented on a black and white CRT monitor (Clinton/Richardson M20DCD1RE Monochrome CRT) with a resolution of 32 pixels/cm. The display was viewed from a distance 215 cm, yielding a visual resolution of 120 pixels/degree of visual angle. The display was controlled by BITS++ digital video processor (Cambridge Research Systems), which interposes a 14-bit look-up-table between the 8-bit digital computer display output (DVI) and the analog monitor. This allowed us to linearize the display and control contrast by loading appropriate LUTs constructed with reference to a previously measured monitor gamma function [14]. The maximum luminance of the monitor was set to 200 cd/m^2^. Observers viewed the display binocularly with natural pupils in an otherwise dark room.

All stimuli were constructed as digital movies using the QuickTime media architecture (Apple Computer). They were displayed using the ShowTime software that we have developed for calibrated presentation of QuickTime movies [15], [16]. Each movie consisted of a sequence of images (frames) presented at a frame rate of 75 Hz. Each movie frame was 256×256 pixels (2.133×2.133 degree) in size. The LUT ensured that the relation between the graylevel G of each pixel and the resulting luminance of that pixel was given by
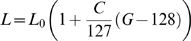
(1)where C is the peak contrast of the image and *L*_0_ is the mean luminance. In these experiments, *L*_0_ = 100 cd/m^2^. The values of *G* ranged from 1 to 255. The peak contrast was used to control both the time course of the stimulus and the overall peak contrast of the movie. From frame to frame, the contrast of each image was varied as a Gaussian function of time, with a standard deviation of 0.125 msec. This time-course was not intended to simulate the real-world time course of an aircraft, but rather was selected to limit the duration of each stimulus, but also to ensure that the target did not appear or disappear abruptly. Other research has shown that this slow onset and offset yields contrast thresholds similar in properties for those of stationary targets [19]. The total duration was 37 frames, or 0.493 sec. One example movie is shown in supporting file Movie S1.

Stimuli were presented at the center of an otherwise uniform gray (*G* = 128) screen. Two sets of fixation marks were provided. The first were dark corner marks (*G* = 1) at each corner of the 256×256 stimulus region. These marks were visible throughout the block of trials, and were the same as those used in a previous experiment from which the SSO was derived [12], [17]. The second type of mark was a fixation point (a cross of size 3×3 pixels, *G* = 0) at the center of the stimulus region. It was present at all times except during the actual stimulus presentation.

### Gabor stimuli

**Figure 2:**
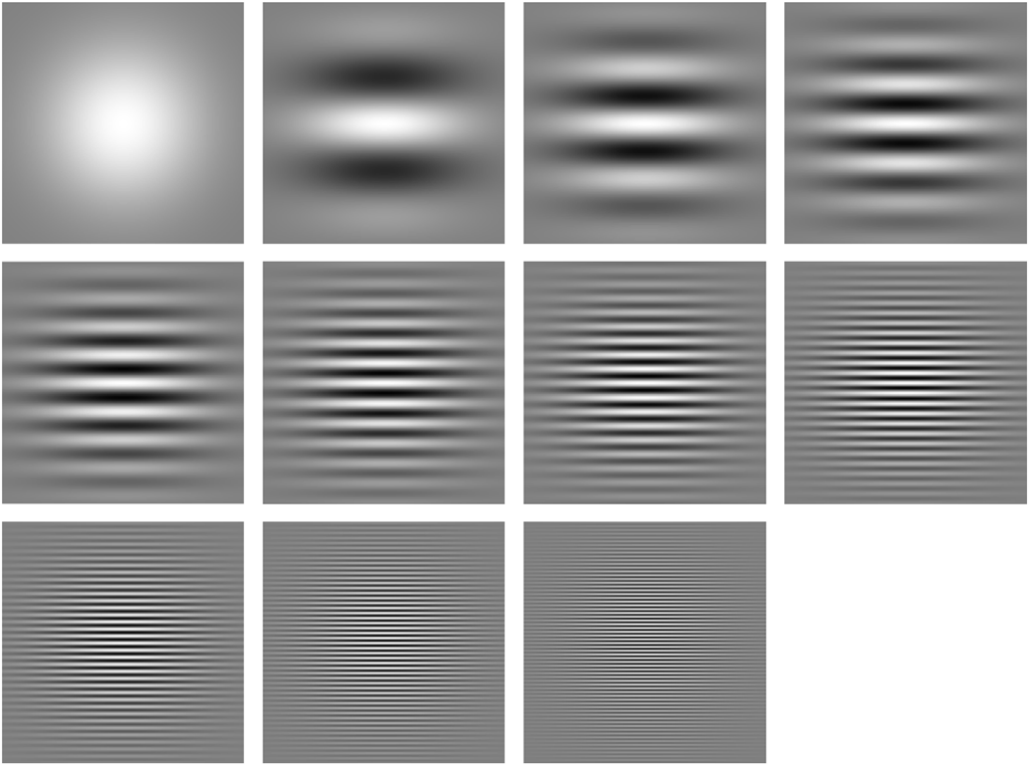
Gabor stimuli. Each is a luminance variation that is the product of a Gaussian and a sinusoid. The Gaussian standard deviation was fixed at 0.5 deg and the frequencies are 0 (pure Gaussian), 1.12, 2, 2.83, 4, 5.66, 8, 11.31, 16, 22.63, and 30 cycles/deg. Each square is 2.133 degree per side.

Two types of stimuli were used: Gabor patterns and rendered images of aircraft. As shown in Figure 2, each of the eleven Gabor stimuli was the product of a Gaussian and a sinusoid. The Gaussian standard deviation was fixed at 0.5 deg and the frequencies of the sinusoids were 0, 1.12, 2, 2.83, 4, 5.66, 8, 11.31, 16, 22.63, and 30 cycles/deg. These Gabor stimuli were identical to those used in the ModelFest experiment, and were in fact extracted from a file available online (http://journalofvision.org/5/9/6/modelfest-stimuli.raw) containing those stimuli [12]. We collected Gabor thresholds in order to compare the performance of our present observers with that of the ModelFest observers, and with the corresponding predictions of the SSO.

### Aircraft stimuli

**Figure 3:**
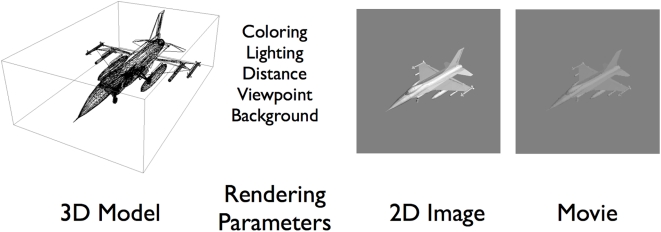
Rendering of aircraft stimuli from 3D graphic models. The first panel shows the 3D graphic model, depicted as a collection of triangular facets. The second panels shows the conditions that determine the rendering of the model. The third panel shows an example of a rendered 2D image. The third panel represents one frame from the stimulus movie created from the rendered 2D image. The corresponding movie is provided as supporting file Movie S1.

**Figure 4:**
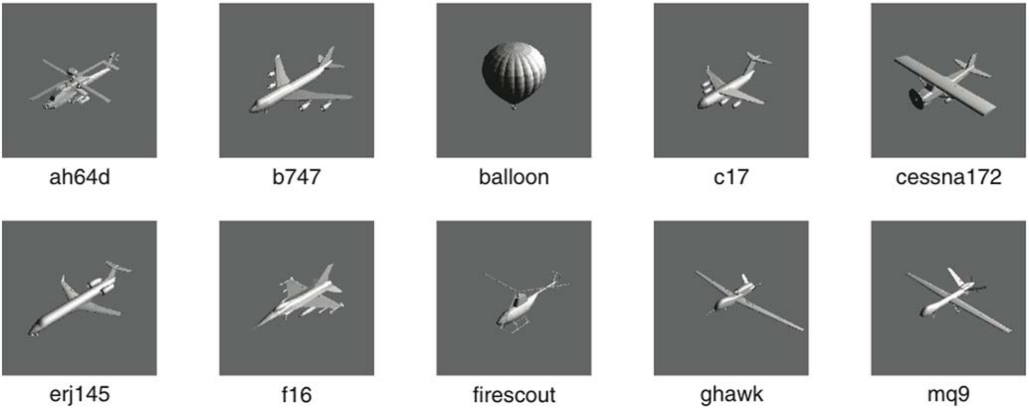
The ten aircraft used in the experiment. A 2D rendered image and short name for each model is shown.

The aircraft images were obtained by rendering three-dimensional graphic aircraft models, as shown in Figure 3. Ten different aircraft were used, as shown in Figure 4. For reference, we introduce brief names for the ten aircraft: ah64d, b747, balloon, c17, cessna172, erj145, f16, firescout, ghawk, mq9. The aircraft were selected to provide a broad cross-section of current vehicles, and to provide a broad range of shapes. All models were purchased on the internet (http://www.amazing3d.com/), and varied in the number of triangles included (a simple measure of the complexity of the model). Aircraft dimensions were obtained from online information provided by aircraft manufacturers or other public sources. Data regarding each model are provided in Table 1.

**Table 1:** Aircraft models and aircraft dimensions in meters.

Name	Aircraft	Triangles	Wingspan	Length	Height
ah64d	AH 64-D helicopter	3,278	14.63	17.73	3.87
b747	Boeing 747	4,426	64.4	70.7	19.4
balloon	Hot air ballooon	16,000	10		
c17	MD C-17	2,450	51.76	53.04	16.79
cessna172	Cessna 172	1,300	10.92	8.21	2.68
erj145	Embraer ERJ-145	7,209	20.04	29.87	6.75
f16	F-16	4,400	9.8	14.8	4.8
firescout	Firescout UAV	2,521	8.38	6.98	2.87
ghawk	Global Hawk	10,773	35.4	13.5	4.6
mq9	MQ-9 Predator	15,924	20.1	11	2.4

To render the model into a two-dimensional image, it was first necessary to specify the lighting conditions, the viewpoint, the viewing distance, and the color of the aircraft surfaces. The aircraft image was rendered against a uniform gray background of graylevel 128. The movie was constructed as for the Gabor stimuli, by setting the contrast on each frame to a Gaussian function of time.

The aircraft images were obtained using the rendering capabilities of the Mathematica programming system [25]. We used Mathematica 5.2 on an Apple Macintosh computer. The rendering was governed by a number of parameters, which we set down here for future reference. Because changes in software versions and platforms cannot guarantee the same result, we also provide a file of all 38 aircraft images used in these experiments.

Each 2D image was rendered by exporting the 3D graphic model to an image file, using a number of graphic options that are listed below in Table 2. Where multiple values of an option are shown, these were used to create alternate versions of the image, for different brightnesses or viewpoints.

**Table 2:** Rendering parameters for aircraft images.

Option	Value 1	Value 2	Value 3
Boxed	False		
SphericalRegion	True		
Format	“PGM”		
ImageResolution	72		
ImageSize	{256, 256}		
	Viewpoint 1	Viewpoint 2	Viewpoint 3
ViewPoint	{0, −1, 0}	{1, 0, 0}	{1, −1, 1}/Sqrt[3]
Lighting	{{“Ambient”, GrayLevel[0.2]}, {“Point”, White, Scaled[{2, 0, 2}]}}	{{“Ambient”, GrayLevel[0.2]}, {“Point”, White, Scaled[{2, 2, 2}]}}	{{“Ambient”, GrayLevel[0.2]}, {“Point”, White, Scaled[{2, 2, 2}]}}
	Bright aircraft	Dark aircraft	
SurfaceColor	GrayLevel[.8]	GrayLevel[.2],	
SurfaceSpecularity	Specularity[.2, 3]	Specularity[0.2, 5]	

Viewing distance was varied using the PlotRange option. It was set, in each dimension, to plus and minus a distance that was in turn specified as a multiple of the radius of a sphere enclosing the aircraft model. In the experiment size was indexed by an integer *k* from 0 to 4. In each case the radius multiplier was 2*^k^* ^+.5^. Thus for an index 0, the viewing distance was 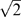 radii.

**Figure 5:**
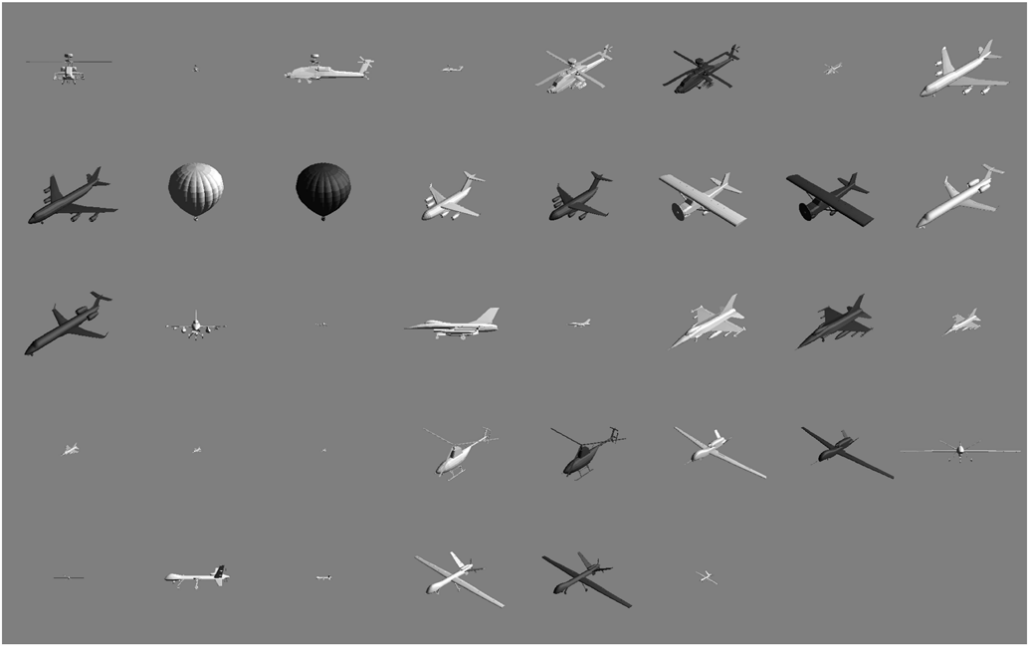
Aircraft images used in the visibility experiment. Aircraft vary in type, size (distance), orientation, and contrast (darker or lighter than background).

The file containing all 38 aircraft images is called WatsonAircraftImages.zip. It is a compressed binary file. After decompression, pixels are represented as 8-bit unsigned integers in row-major order. The images are in alphabetical order of their symbolic names, which are of the form c-v-s-b, where c = craft name, v = viewpoint (1, 2, 3), s = size (0, 1, 2, 3, 4), and b = brightness (1, 2). The complete set of symbolic names, and the order in which they appear in the file, is: {ah64d-1-0-1, ah64d-1-2-1, ah64d-2-0-1, ah64d-2-2-1, ah64d-3-0-1, ah64d-3-0-2, ah64d-3-2-1, b747-3-0-1, b747-3-0-2, balloon-3-0-1, balloon-3-0-2, c17-3-0-1, c17-3-0-2, cessna172-3-0-1, cessna172-3-0-2, erj145-3-0-1, erj145-3-0-2, f16-1-0-1, f16-1-2-1, f16-2-0-1, f16-2-2-1, f16-3-0-1, f16-3-0-2, f16-3-1-1, f16-3-2-1, f16-3-3-1, f16-3-4-1, firescout-3-0-1, firescout-3-0-2, ghawk-3-0-1, ghawk-3-0-2, mq9-1-0-1, mq9-1-2-1, mq9-2-0-1, mq9-2-2-1, mq9-3-0-1, mq9-3-0-2, mq9-3-2-1}. This is also the order in which the images appear in Figure 5.

This file can be read into an array of images using the following Mathematica command:

images = Fold[Partition, Import[“WatsonAircraftImages.zip”, “Byte”], {256, 256}];

In the experiment, the rendered aircraft images varied in size, orientation, and contrast polarity. Sizes varied in steps of a factor of two over a range of a factor of 16. The three orientations were: head on, from the side, and from an oblique direction. The two contrast polarities were positive (aircraft brighter than background), and negative (aircraft darker than background). The complete collection of 38 aircraft stimuli is shown in Figure 5.

### Data collection

Contrast thresholds were measured in a block of 32 two-interval forced-choice trials in a Quest adaptive procedure [18]. The two presentation intervals were marked by tones, and feedback was provided after the observers response. Responses were made using a game controller (Mad Catz. http://www.madcatz.com/). The sequence of trials in each block was converted to a contrast threshold by fitting the percent correct with a Weibull function [19], [20] and locating the 82% correct point. Each block of 32 trials contained only a single stimulus. Stimuli were divided into five sets: 1) Gabors (n = 11), 2) all aircraft at the largest size with positive contrast (n = 10), 3) all aircraft at the largest size with negative contrast (n = 10), 4) one aircraft (f16) at five different sizes (n = 5), and 5) three different viewpoints for three different aircraft at two different sizes (n = 18). The total number of images is 38 (the sets overlap). Within each set, a block of trials was run for each stimulus in random order. This was repeated three times, with a new random sequence each time. Thus three replications of each threshold were obtained for each stimulus and observer.

Each block took about four minutes to complete. Observers were encouraged to rest between blocks, and no more than ten blocks were completed by a single observer on a single day. Experimental protocols were approved by the Human Research Institutional Review Board of NASA Ames Research Center.

### Observers

Three observers took part in the experiment: ABW, a 56 year old male, CVR, a 36 year old male, and ES, a 30 year old female. ABW wore a recent spectacle correction. Immediately prior to the experiment, CVR and ES were examined by an optometrist and assigned a new spectacle correction (ES) or no correction (CVR). All three are experienced observers; only ABW (the author) was aware of the purposes of the experiment at the time of data collection.

## Results

In most of the figures that depict basic contrast threshold results, we show contrast on the vertical log scale, and use a fixed range extending from 0.003 to 0.8. This range is large enough to include all of our data, and the use of a fixed scale, while wasting some amount of space on the page, allows a more consistent and direct comparison of data from the several figures.

### Gabor Stimuli

**Figure 6:**
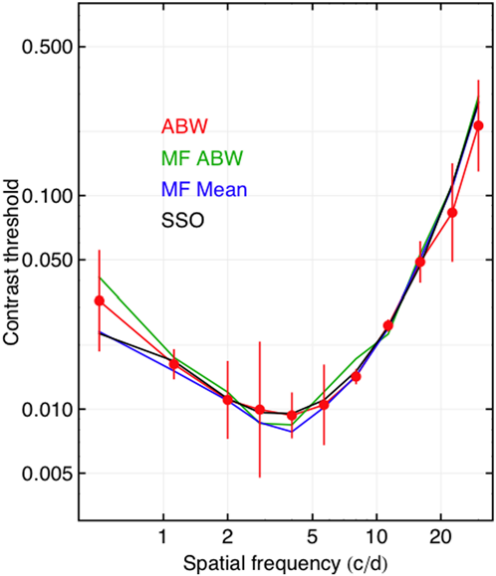
Contrast thresholds for Gabor stimuli for observer ABW from the present experiment (red), along with ABW data from the original ModelFest experiment (green), the mean data from the ModelFest experiment (blue), and the predictions of the Spatial Standard Observer (black). The error bars are plus and minus one standard deviation. The lowest frequency point is for 0 cycles/deg (a simple Gaussian, see Figure 2).

The experimental methods used here, and the Gabor stimuli, and one of the observers (ABW) were identical to those used in the ModelFest experiment. This enables a direct comparison of the results from the two experiments. In Figure 6 we plot the contrast thresholds for Gabor targets for observer ABW from the present experiment (red), along with three measures derived from the ModelFest experiment. These are: the ModelFest data of observer ABW (green), the mean of 16 ModelFest observers (blue), and the predictions of the SSO model (black), that was derived from the ModelFest data.

The close agreement among all four curves suggests that we have closely duplicated the conditions of the ModelFest experiment, and that the sensitivity of observer ABW has not changed markedly since the ModelFest data were collected (1999). It also indicates that for this observer, at least, the SSO model continues to provide a good description of the data.

**Figure 7:**
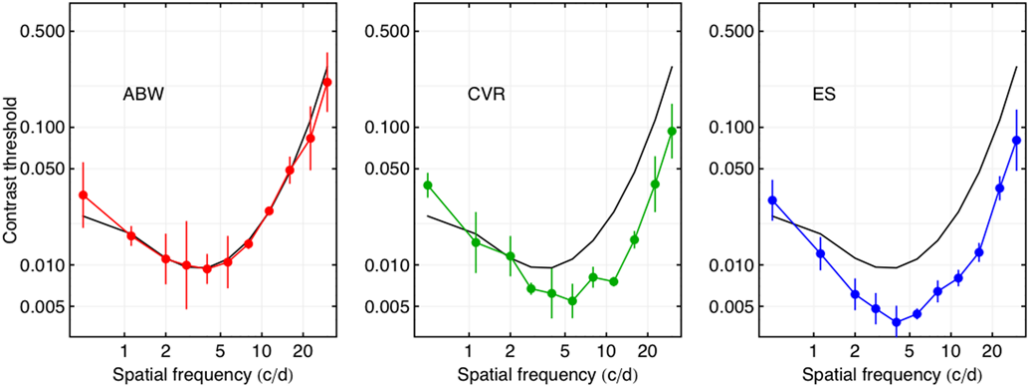
Contrast thresholds for Gabor targets for three observers. The black curve in each panel is the predictions of the SSO. The error bars are plus and minus one standard deviation. The lowest frequency point is for 0 cycles/deg (a simple Gaussian, see Figure 2).

In Figure 7 we plot the data for all three observers, along with the SSO predictions. It is evident that observers ES and CVR are considerably more sensitive at mid to high frequencies than either ABW or the SSO model. They have lower minimum thresholds, and they are more acute (they can see higher spatial frequencies). However, the general shape of the results is consistent among observers. This outcome suggests that the SSO model may underestimate sensitivity and acuity for young, well-corrected observers.

Another discrepancy between the present data, for all observers, and the SSO model is the behavior at the lowest frequency. Though plotted at 0.5 cycles/deg, this is the 0 cycle/deg Gabor, or simple Gaussian (the first target in Figure 2). For all three observers this lies above the model prediction, and lies on a trajectory smoother and more upward going than the SSO. This behavior is also evident in the ModelFest data for observer ABW (green curve in Figure 6). This may be due to an artifact in the method used by some laboratories in the ModelFest experiment [17], [21], which may in turn have distorted the shape of the SSO CSF at the lowest frequencies. In brief, the ModelFest mean observer, and the SSO, may overestimate sensitivity to the lowest spatial frequencies.

The purpose of collecting threshold for Gabor targets in this experiment was twofold: to measure the contrast sensitivity function for each observer, and to verify that they agreed with the predictions of the SSO. The results indicate that the SSO is well matched to the CSF of observer ABW, but underestimates sensitivity of observers ES and CVR in systematic ways. Since the SSO is intended to mirror a population average, it cannot be expected to agree with the results of every observer. But the discrepancy between model and observers should be borne in mind as we look at subsequent predictions for these observers. Later, we will show how the SSO can be adjusted to better match the results of our two more sensitive observers.

### Aircraft Stimuli

**Figure 8:**
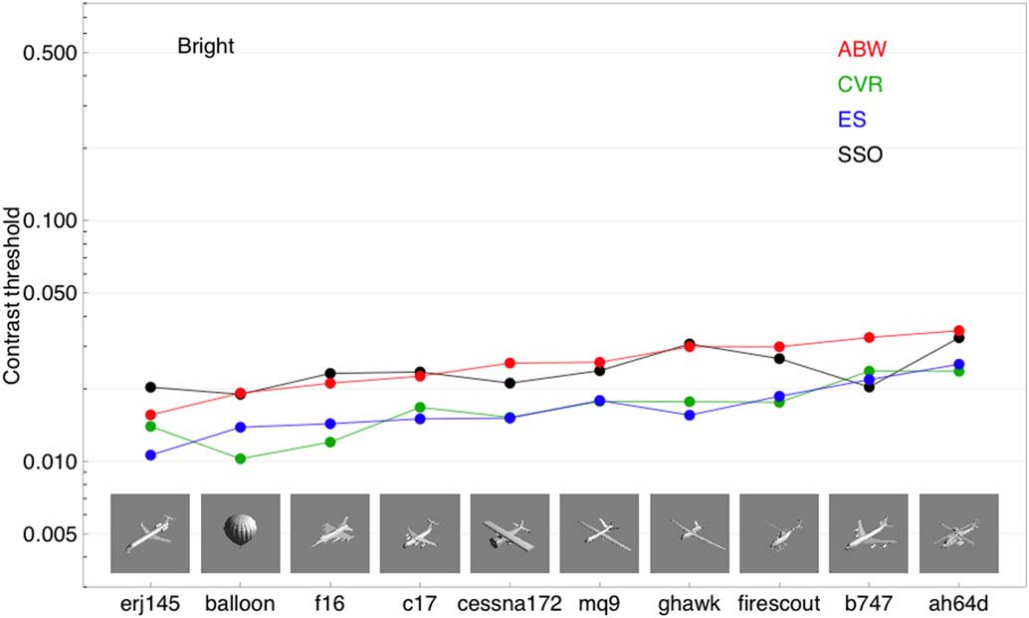
Contrast thresholds for three observers for ten different aircraft. All the aircraft have positive contrast (are brighter than the background). Actual aircraft images are shown in the figure. The black points are SSO predictions. The data are sorted from left to right according to the mean threshold for the three observers.

Figure 8 plots contrast thresholds for individual aircraft targets of the largest size. We selected this size because we believed that differences in visibility based on shape would be most evident at the largest sizes. Pictures of the targets are shown within the graph. The figure also shows the SSO predictions. The horizontal positions of the aircraft are in order of the mean threshold for the three observers. Note that the trend for each observer is similar or identical to the mean trend. This indicates that there are systematic differences between thresholds for the different aircraft. The range of mean values spans a factor of 2.08. However, relative to the range of thresholds for Gabor targets, these differences are small.

The data for observer ABW lie about a factor of 1.5 above the data for ES and CVR, consistent with the differences in sensitivity seen in the Gabor target data (Figure 7). Note also that the data for ES and CVR are very close to each other.

Examination of the SSO predictions shows they agree remarkably well with the data of ABW, but are systematically above the data for ES and CVR, again consistent with the results for Gabor targets, and our earlier observations about the fit of the SSO model. The general conclusion is that the SSO accurately predicts the variations in visibility across aircraft type. Note that this range includes a wide variation in shape, from a nearly circular uniform target like “balloon” to a highly elongated form such as “ghawk.” Note also that these SSO predictions have no degrees of freedom or adjusted parameters.

**Figure 9:**
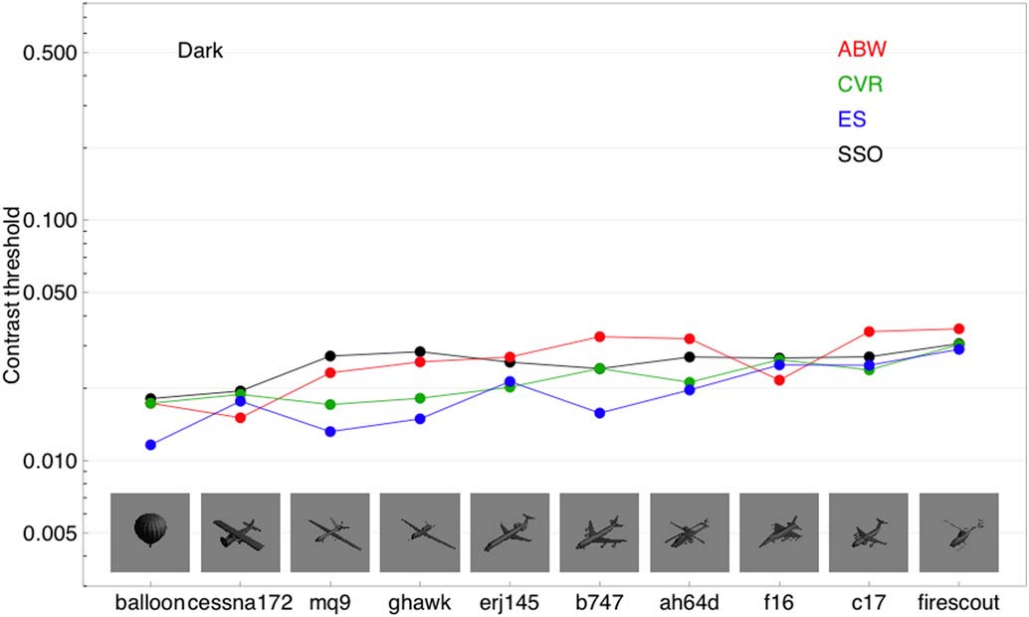
Contrast thresholds for three observers for ten different aircraft. All the aircraft have negative contrast (are darker than the background). Actual aircraft images are shown in the figure. The black points are SSO predictions. The data are sorted from left to right according to the mean threshold for the three observers.

Figure 9 shows corresponding data for the dark aircraft at the largest size. These have also been sorted left to right according to mean threshold. The order is similar but not identical to that for the bright aircraft. For the dark stimuli, the results of ES and CVR are not as clearly separated from those for ABW. Here again the SSO predictions are quite good for ABW, and reasonably good for the other two observers.

**Figure 10:**
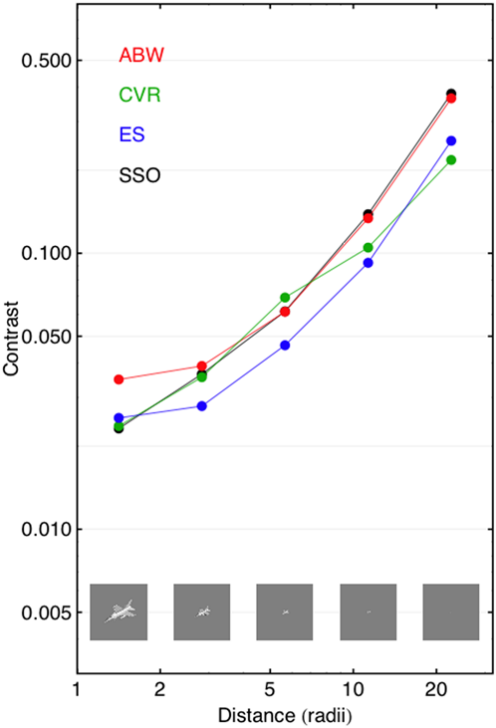
Contrast thresholds for aircraft targets varying in size. Results from three observers and predictions of the SSO are shown. Actual aircraft images are shown in the figure.

In Figure 10 we plot threshold as a function of size, or its inverse, distance, for one aircraft (f16). Here we see that size, unlike shape, produces very large changes in visibility (a factor of 9.9 from highest to lowest average threshold). Again, the data for ES and CVR are very similar to those for ABW, but shifted to slightly lower thresholds.

Again, there is a remarkably good agreement between the ABW data and the SSO predictions, except at the largest size. This discrepancy is consistent with the deviation of the SSO from the ABW Gabor data: large aircraft targets are dominated by low spatial frequencies, and at low frequencies the SSO overestimates sensitivity. The data for the other two observers are also predicted well by the SSO, except for an expected vertical shift.

**Figure 11:**
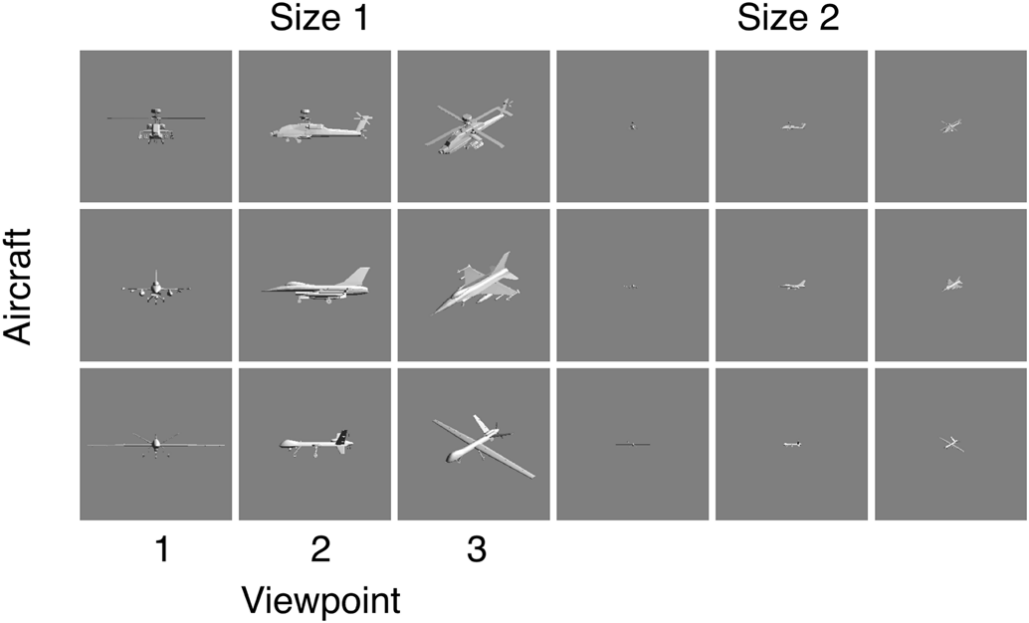
Images of aircraft varying in orientation, size, and type. These images were used to test the effect of viewpoint on the contrast threshold.

**Figure 12:**
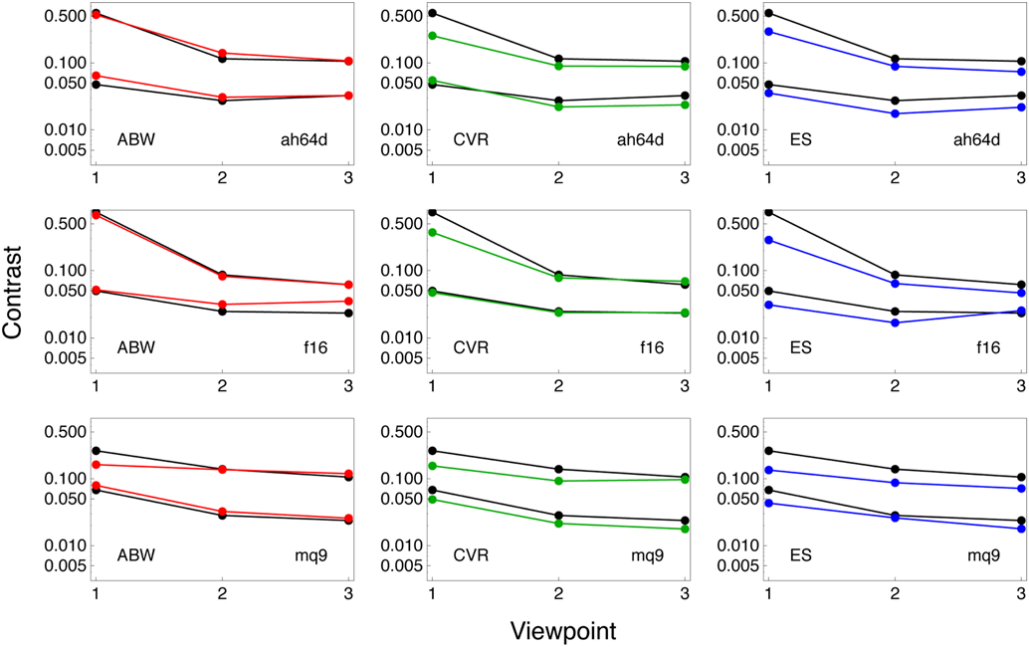
Contrast thresholds for three viewpoints of three aircraft at two sizes for three observers. SSO predictions are also shown in black. Each panel shows the data for one observer and one aircraft. The two colored curves in each panel are for the two sizes. The horizontal axis indicates the orientation of the aircraft. The stimuli for these data are shown in Figure 11.

To examine variations in threshold due to the orientation of the aircraft relative to the viewer, we used three types of aircraft (ah64d, f16, and mq9) at two sizes (0 and 2). Each of these was rendered in three orientations: head on, from the side, and from an oblique direction. These eighteen conditions are shown in Figure 11. In Figure 12 we plot the contrast thresholds for these eighteen conditions for our three observers. Individual panels show the data for one observer and one aircraft. The abscissa of each panel indicates the three orientations, while the two curves in each panel are for the two different sizes. As usual, the SSO predictions are depicted in black.

As expected, there is a pronounced effect of size (two curves of the same color in each panel). There is also an effect of orientation, with the head-on view (1) having a higher threshold than the other two views. As in the previous figures, the SSO provides a good description of these variations. The fit is best for ABW, but the deviations for the other two observers are primarily a vertical shift consistent with their higher sensitivity. They also show a smaller elevation at the smallest size with viewpoint 1, which is consistent with their higher acuity.

### Comparison of Data and SSO

**Figure 13:**
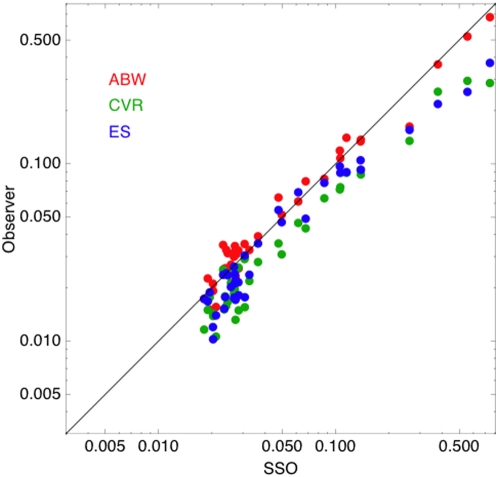
Human observer contrast thresholds versus SSO predictions for all aircraft targets.

In the previous sections and figures we have seen that the SSO provides a good account of the contrast thresholds for a range of aircraft images. It correctly mirrors the variations in threshold due to aircraft type, size, and orientation, as well as interactions among these variables. Here we summarize those results by looking at all 38 aircraft thresholds for each of the three observers. In Figure 13 we plot each threshold against the SSO prediction for the same target. The observers are depicted by the standard colors. Accurate predictions would fall along the line of unit slope. As can be seen, the points for observer ABW are very close to this line. The results for the other two observers follow a similar pattern, but fall below the line. This is consistent with our earlier observation that observers CVR and ES are more sensitive and more acute than observer ABW or than the SSO. For reference, the RMS errors between data and model for ABW, CVR, and ES, in log units, are 0.077, 0.187, 0.152, respectively.

**Figure 14:**
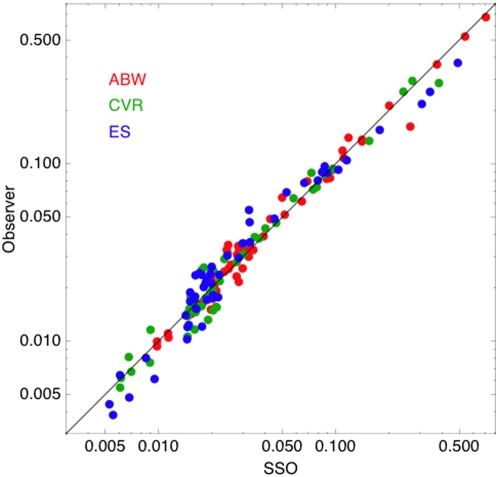
Human observer contrast thresholds versus SSO predictions for both aircraft and Gabor targets. The SSO parameters were re-estimation for each observer to optimize the fit.

To this point in our analysis we have used the default parameters of the SSO model. The parameters of the SSO govern the shape of the contrast sensitivity function, and the nature of the pooling over space. For further details on these parameters consult [12]. To evaluate the effect of a misfit between the SSO and our observers, we fit the SSO to both Gabor and aircraft thresholds, allowing the seven parameters of the SSO to vary till they provide the minimum squared error for each of the three observers. The results are shown in Figure 14.

The points lie close to the unit-slope line, indicating a good fit for each observer. For reference, we provide the old and new parameters in Table 3, along with residual RMS errors. Observer ABW, as expected, shows only a small reduction in RMS error, while the other two observers show much larger improvements. As expected, observers CVR and ES show substantial increases in sensitivity (Gain) and acuity (*f*_0_) relative to either observer ABW or the default SSO.

**Table 3:** SSO parameters before and after estimation from present data.

Observer	RMS	Gain	*f*_0_	*f*_1_	*a*	*ρ*	*σ*	*β*
SSO		373.083	4.17263	1.36254	0.849338	0.778593	1.57237	2.4081
ABW	0.0664	0.679	1.035	0.889	1.052	0.959	2.111	1.076
CVR	0.0731	1.090	2.416	1.013	1.106	1.520	0.941	0.903
ES	0.0975	1.991	1.501‵	1.520	1.107	1.128	0.854	0.780

Original parameters are given for observer “SSO.” For the three human observers, we show the ratio of new and old parameters.

**Figure 15:**
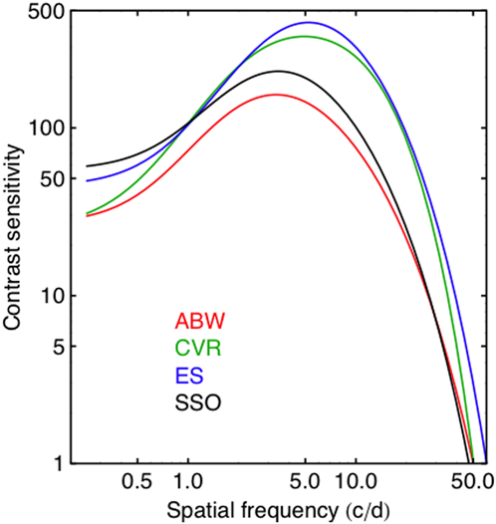
Contrast sensitivity functions derived from SSO parameters optimized for each observer. The black curve is the original SSO CSF. For two of the observers, the curves lie above and to the right of the original curve, indicating that they have greater sensitivity and acuity than the SSO.

For comparison, we show in Figure 15 the contrast sensitivity functions for the three observers derived from the new fits, along with the default SSO function. It is evident that the functions for ES and CVR show greater sensitivity and acuity than the SSO.

## Discussion

### Fit of data and model

In general, the SSO provides a good overall prediction of contrast thresholds for aircraft images varying in aircraft type, size, contrast polarity, and orientation. The predictions are excellent for one observer, and slightly under-predict sensitivity for the two other observers. When model parameters are adjusted to match observers sensitivity and acuity, all predictions are excellent.

### Observer differences

It is clear that two of our observers (ES and CVR) are more sensitive and more acute than ABW. This is perhaps related to their younger age, and up-to-date refraction. It also seems possible that the ModelFest population, and by extension the SSO, underestimates the sensitivity of young emmetropic observers.

### Model adjustments

Two of our three observers are more sensitive and more acute than the SSO. Likewise, there are indications that the SSO may overestimate sensitivity to very low frequencies. Finally, measurement of the spatial MTF of the display reveals that a significant part of the attenuation of high spatial frequencies measured in the ModelFest experiment, and incorporated in the SSO, is due to the display rather than the observer (see Appendix C). This suggests the possibility of adjustments to the SSO to better represent the performance of young emmetropic observers, viewing high-resolution displays or real world images. We leave those adjustments for a future project.

### Computing threshold range

One goal of this research is to provide technology and tools that will enable easy and accurate calculation of the maximum distance at which an aircraft will be visible to the human eye: the threshold range. We have seen that the SSO provides predictions that are highly accurate for one observer and only slightly less accurate for the other two observers. This validates the use of the SSO to calculate threshold range estimates, as we now describe.

To compute threshold range values in real world units (meters), we need to relate our stimulus image dimensions to corresponding real world dimensions. First we note that the angular subtense of our stimulus images is 256/120 = 2.133 degrees. Recall that the nominal viewing distance from each aircraft, for purposes of rendering, was specified in units of the radius of the sphere enclosing the aircraft. The aircraft images are designed such that when the distance is one radius, the wingspan of the aircraft approximately spans the image. Since we know the wingspan of each aircraft in meters, this enables us to compute the simulated viewing distance in meters. At a rendering distance of *D* radii, the angular subtense is
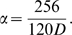
(2)In the real world, this same angle would be subtended by a wingspan of *W* meters at a range *R* meters when
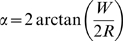
(3)Solving for *R*, we have
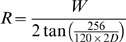
(4)Using the small angle approximation tan(x) = x/57.296,
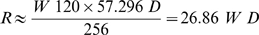
(5)


Note that the predictions in Figure 10 give contrast threshold as a function of distance in radii. We can re-plot those values as radii, converted to meters by Equation 4, as a function of contrast. And we can do this not just for the aircraft in Figure 10 (f16), but for all ten of our aircraft. As the target becomes very small, the accuracy of the rendering becomes problematic. Accordingly, these predictions were made by using a fixed large rendering of the aircraft (as in Figure 4), and varying the imputed scale of the image for the standard observer model. The side-on view was used.

**Figure 16:**
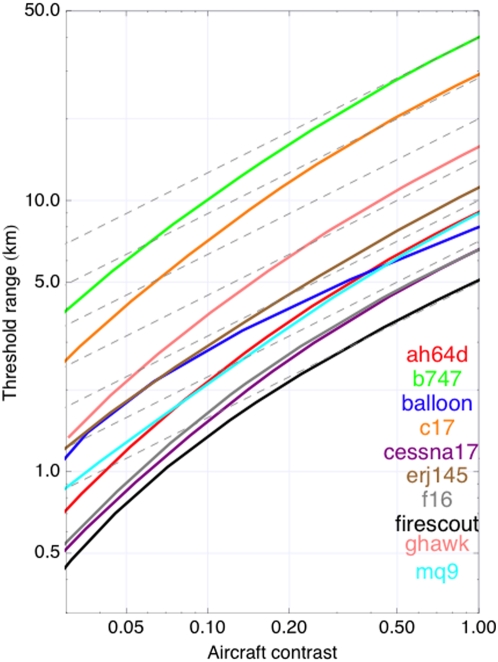
Threshold range as a function of contrast for ten aircraft. Threshold range is the largest distance at which an aircraft can be seen. Dashed lines have slope = ½. These results depend upon a particular set of lighting conditions.

The result of these calculations is shown in Figure 16. The figure shows, for each aircraft at a given contrast, the range at which it can be seen. We must caution that these predictions are for a single brief exposure, and are for the specific view and illumination conditions considered. As will be discussed below, other conditions may yield threshold ranges larger or smaller than these values.

The figure shows that range increases markedly with contrast. The dashed lines have a slope of one half, which is the expected relationship when threshold is determined by the integrated contrast. Most of the craft approximate this slope at the highest contrasts (which correspond to the smallest sizes), but depart markedly from it over the full range of contrasts. The different aircraft lie at different vertical positions, due largely to their different absolute sizes. This figure illustrates an obvious but important point. It is not possible to know the visible range for an aircraft without knowing its size, shape, orientation, brightness, and the brightness of the background sky. It is clearly impractical to make real world empirical measurements of visible range for all of these variables. That is why a tool such as the SSO is of value in this situation. It can calculate the result over any distribution of desired parameters, and can do so without the effort and expense of field tests.

#### Comparison with Howell data

**Figure 17:**
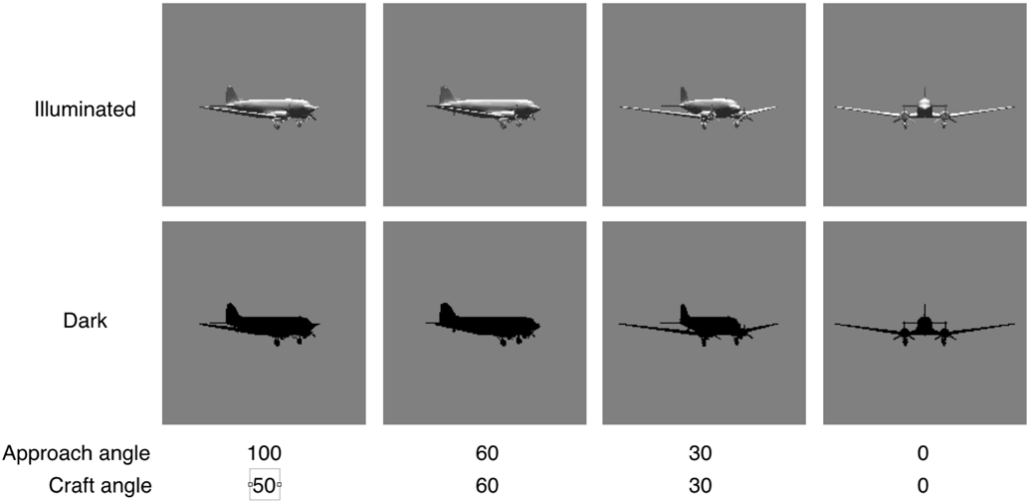
Rendered images of DC-3 aircraft at four approach angles and two illumination conditions. These are the approach angles used in the experiment of Howell [5].

As discussed in the introduction, Howell [5] measured the “threshold distance” at which an aircraft (DC-3) could be detected by a vigilant observer precisely aware of the direction of approach. We can now compare the predictions of the SSO to these data. To achieve this, we secured a 3D graphic model of the DC-3 aircraft, and generated range predictions as described above. In Howell's experiment, the intruder aircraft approached the subject aircraft from four different angles: 0°, 30°, 60°, and 100°. These correspond to rotations of the view of the intruder aircraft of 0°, 30°, 60°, and 50°. We computed separate predictions for these four approach angles. In his report, Howell noted that the greatest threshold distances occurred for negative contrast (dark aircraft against a bright sky). Accordingly we generated predictions for two illumination conditions: one in which the sun was above the subject aircraft, and the other in which there was no sun illumination at all. The latter case yields a black silhouette against a light background, which is the extreme case of negative contrast. The rendered aircraft images for these various conditions are shown in Figure 17.

**Figure 18:**
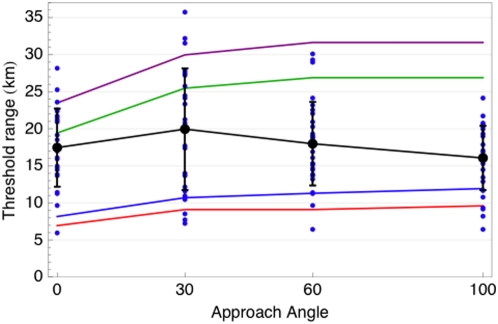
Threshold distance for a DC-3 aircraft at four viewing angles. The points are data from Howell [5], and the black lines show mean and plus and minus one standard deviation. The red curve is the SSO prediction for a well-lit craft and the green curve is SSO prediction for a dark silhouette. The blue and purple curves are comparable predictions for a highly sensitive observer (ES).

In addition, noting the superiority of two of our observers to the SSO, both in sensitivity and acuity (see Figure 15), we also generated predictions for our most sensitive observer ES. Our range predictions, along with the data of Howell, are shown in Figure 18.

Our predictions for the two illumination conditions differ by almost a factor of three, and it should be clear that, depending on environmental conditions and aircraft coloration, even larger differences can be expected. These variations ultimately effect the contrast, and hence the predicted range. Howell, commenting on the very large variability in his results, attributes it largely to this cause. The upper curves, for silhouette targets, are a relatively fixed upper bound, but the lower curves are highly dependent on the specific environmental conditions. With these caveats in mind, we can say that our various predictions evidently bracket Howell's data. The data show some effect of angle, but the large variability obscures the significance of these effects. Our predictions mirror the decline in range at the 0° approach angle, due presumably to the smaller profile, but do not show the observed small decline at the two largest angles.

Additional caveats regarding these predictions should be mentioned. We have assumed a meteorological range of infinity (no atmospheric attenuation of contrast) so our predictions are an upper bound. On the other hand, our predictions are for a very brief presentation (∼1/4 sec), while the real world target persists until detected. Visibility rises slowly (approximately fourth root) with duration following the first quarter second [19], but extending the duration would nonetheless elevate our predictions somewhat. For example, if the observer could maintain fixation on the target for a full second, that might lower contrast threshold by a factor of √2. From the curves in Figure 16, we see that this would yield an increase of range of only about 25%.

#### Limitations

First, as noted in the introduction, we have not measured or predicted the visibility of aircraft against patterned backgrounds. Uniform backgrounds provide a best case, with respect to visibility and range, and are thus a critical first step. But in the future we hope to employ the masking capability of the SSO to include patterned backgrounds as well. We should note that a patterned background may introduce two fundamental visual problems: masking and relative motion. The latter problem, in particular, may require attention to the time domain, which we have largely ignored in this report. We speculate that cloud backgrounds, because they are dominated by low spatial frequencies with low contrast, do not cause much masking or relative motion, but that terrain backgrounds will produce both masking and relative motion.

A second limitation is that we analyze and predict thresholds for photopic (daylight) vision only. At dimmer illuminations, the contrast sensitivity function (e.g., Figure 7) moves lower and to the left, which will alter the predicted thresholds. Nonetheless, these new thresholds could be predicted with a suitable change in model parameters. Nightime conditions may introduce a rather different challenge: understanding the visibility of warning lights, which we do not deal with here.

Second, as noted in the discussion of prior research, the challenge of locating an aircraft in the sky depends upon contrast detection, as modeled here, but also upon the process of visual search, which we have not modeled. (This is why we have compared our predictions to the data of Howell, in which the location of the approaching aircraft was known.) We can be certain, however, that search cannot succeed without contrast detection; thus our model provides a lower bound on the contrast required, or an upper bound on the distance at which detection is possible. A model of visibility, like that provided here, is an essential component of any model of visual search.

#### Extensions

We have demonstrated the validity and utility of the SSO as a tool for computing visibility range. This utility can be extended in a number of ways. Elsewhere, we have shown how the SSO can be extended with an optical module that can simulate any specified set of waveform aberrations of the eye [22]. This would allow us to simulate a pilot or ground observer, or a population of such individuals, with specified optical limitations. Since age has somewhat predictable effects on visual sensitivity [23], [24], it would be possible to observers of a specific age as well.

Because the SSO operates on images, the limitations of the viewing system can be incorporated into the calculations. For example, the effects of remote viewing through a low-resolution video channel with limited dynamic range could be simulated. This would allow simulation of the viewing conditions for the UA pilot.

The visibility range predictions in Figure 16 do not take into account the diminution in contrast due to attenuation by the atmosphere. This effect can of course be small or large, depending on atmospheric conditions. However, there are already well-developed models for these atmospheric effects, which can calculate the reduction in contrast of the aircraft image at a specified distance under specified atmospheric conditions [6], [7]. It is a relatively simple matter to incorporate such an atmospheric model into the detection range calculations of the SSO.

#### Display MTF

**Figure 19:**
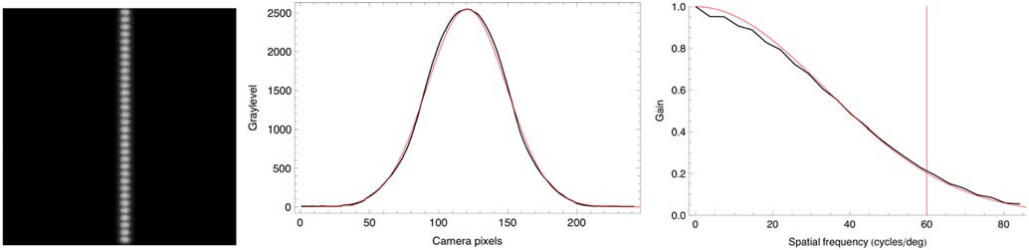
Estimation of display MTF. A) Captured image of a vertical line on the CRT display (graylevel = 255). B) The linespread function obtained by averaging the image over columns of pixels. The red curve is a Gaussian fit. C) MTF obtained as the Discrete Fourier Transform of the linespread function. The red curve is a Gaussian fit.

In order to better understand the rendition of our aircraft images on our display we measured the modulation transfer function (MTF) of the CRT. This was achieved by capturing digital photographs of horizontal and vertical single pixel wide lines, and averaging over columns or rows, respectively, to obtain the linespread function. The magnitude of the Fourier Transform of the linespread function is an estimate of the modulation transfer function (MTF) of the display, which describes the attenuation of contrast of sinusoids of various frequencies. We fit each MTF with a Gaussian to provide a summary measure of the MTF. We repeated this exercise at two line graylevels (128 and 255) on a black (0) background, because the CRT spot size is known to grow with brightness, which will alter the MTF. One case is shown in Figure 19.

The figure shows that the display imposes considerable attenuation of visible spatial frequencies in the horizontal dimension. Similar results, with somewhat less attenuation, were found in the vertical dimension. A summary of Gaussian scale parameters for the four cases (two orientations and two graylevels) is shown in Table 4. The significance of these results is that they show that both this experiment, and the ModelFest experiment (which used a CRT for every observer and this particular CRT for three observers) are likely to underestimate the acuity of observers. Likewise the aircraft targets, particularly the small ones, did not have as much contrast as their digital representations would suggest, because they would be attenuated by the display.

**Table 4:** Estimated Gaussian scale parameters for the CRT display.

	128		255	
	Space	Frequency	Space	Frequency
Horizontal modulation	103.177	107.514	84.8073	84.238
Vertical modulation	132.901	125.103	106.142	102.385

Values are shown derived from fits to the linespread and to the MTF, and for grayscales of 128 and 255. The scale is the frequency in cycles/deg at which the Gaussian declines to *e* ^−**π**^ = 0.0432.

It is important to note that the ModelFest experiment, this experiment, and the SSO are all mutually consistent; their only shared flaw is that they all misattribute some attenuation to the observer that should properly be attributed to the display. This has implications for future, more accurate versions of the SSO, and for future psychophysical experiments.

### Conclusions

1. Contrast thresholds were collected from three observers for 38 different images of aircraft superimposed on a uniform gray background. The aircraft differed in type, orientation, distance, and brightness.
2. Contrast thresholds were collected from the same three observers for eleven Gabor targets.
3. Predictions for both Gabor and aircraft targets were computed by the Spatial Standard Observer (SSO) model.
4. Gabor target thresholds were predicted well for one observer, while the other two observers showed greater sensitivity and acuity than the SSO.
5. With adjustments for the greater sensitivity of two observers, aircraft predictions for all three observers were excellent.
6. Threshold range can be computed by the SSO for arbitrary aircraft and viewing conditions.

## Supporting Information

Movie S1 Movie showing an example of a Gabor stimulus with a Gaussian timecourse.
(3.93 MB MOV)
