# Correction: Predicting Visibility of Aircraft

**DOI:** 10.1371/annotation/be07af21-d5b4-4cb3-b311-a3fc275cd9aa

**Published:** 2010-07-15

**Authors:** Andrew Watson, Cesar V. Ramirez, Ellen Salud

There is an error in the authors' affiliation. The Affiliation should be: NASA Ames Research Center, Moffett Field, California, United States of America 

